# Unsafe sexual practice and associated factors among adolescents in secondary schools in Gambella town, Ethiopia: a health belief model

**DOI:** 10.1186/s12889-026-27466-2

**Published:** 2026-04-22

**Authors:** Tariku Frissa, Asrat Zewdie, Dereje Oljira Donacho, Benti Negero

**Affiliations:** 1 Department of Public Health, College of Health Science, Mattu University, Mattu, Ethiopia; 2 Department of Health Informatics, College of Health Science, Mattu University, Mattu, Ethiopia; 3 Department of Midwifery, College of Health Science, Mattu University, Mattu, Ethiopia

**Keywords:** Unsafe sexual practice, Secondary school, Gambela, Health Belif model

## Abstract

**Background:**

Unsafe sexual practices among adolescents represent a significant public health challenge due to their association with increased risk of sexually transmitted infections, including HIV, and unintended pregnancies. The burden remains substantial, contributing to adverse health outcomes, educational disruption and long-term socio-economic consequences. Multiple factors like inadequate sexual health knowledge, limited access to youth-friendly services, low utilisation of preventive methods, and social and behavioural determinants such as peer pressure, substance use, early sexual initiation and weak parental communication further exacerbate the problem.

**Methods:**

An institution-based cross-sectional survey using a self-administered questionnaire was administered to 621 students who were selected using the systematic sampling technique. Unsafe sexual practice was measured using the self-reported behavioural indicators, including inconsistent condom use, multiple sexual partners, and early sexual initiation. The Health Belief Model (HBM) was used to assess psychosocial determinants of these behaviours. The HBM constructs (perceived susceptibility, perceived severity, perceived benefits, perceived barriers, self-efficacy, and cues to action) were measured using validated Likert-scale items. Composite scores were generated for each construct and analyzed to determine their association with unsafe sexual practices. Corresponding p-values and adjusted odds ratios with 95% confidence intervals have been added to improve clarity and statistical interpretation.

**Results:**

The prevalence of unsafe sexual practices was 36.6% [95% CI: 32.8%–40.4%]. The study found that male sex (AOR = 2.38; 95% C.I.: 1.61–3.51, *p* < 0.001), age less than 18 years (AOR = 0.65, 95% C.I.: 0.43–0.97, *p* = 0.038), low perceived norms for abstinence [AOR = 0.6, 95% C.I.: 0.41–0.89, *p* = 0.013], low perceived norms for consistent condom use [AOR = 2.05, 95% C.I.: 1.39–3.03, *p* < 0.001], low perceived self-efficacy for condom use [AOR = 0.59, 95% C.I.: 0.40–0.86, *p* = 0.007], watching sex films [AOR = 1.5, 95% C.I.: 1.01–2.20, *p* = 0.047], chewing khat [AOR = 2.5, 95% C.I.: 1.57–4.07, *p* < 0.001], and drinking alcohol [AOR = 2.93, 95% C.I: 1.86–4.62, *p* < 0.001] were significantly associated with unsafe sexual practice among adolescents.

**Conclusion:**

The study revealed that about two in five students engage in unsafe sexual practices, influenced by socio-demographic and behavioural factors, highlighting the need for collaboration between key stakeholders to mitigate risks and consequences.

**Supplementary Information:**

The online version contains supplementary material available at 10.1186/s12889-026-27466-2.

## Background

Adolescence, defined by the World Health Organization as the period between 10 and 19 years of age, is a critical developmental stage characterized by rapid physical, psychological, and social changes that may predispose young people to risky behaviors [1]. Unsafe sexual practices—such as early sexual debut, inconsistent or non-use of condoms, multiple sexual partners, and engagement with high-risk partners—remain a major public health concern among adolescents, particularly in developing countries [1, 2]. These behaviors substantially increase the risk of sexually transmitted infections (STIs), including HIV/AIDS, unintended pregnancy, unsafe abortion, and their associated social, educational, and economic consequences [2].

Globally, adolescents constitute about 20% of the population, with nearly 90% living in low- and middle-income countries [3]. Sub-Saharan Africa bears a disproportionate share of the burden of adverse sexual and reproductive health outcomes among adolescents, including high rates of HIV infection, early pregnancy, and school dropout [4, 5]. In this region, adolescents and youths account for approximately 20–30% of the total population, making unsafe sexual practices a significant public health and development challenge [5]. Secondary school students, in particular, are vulnerable due to experimentation, peer pressure, limited life experience, and a perceived sense of invulnerability [5].

Evidence from African countries shows that secondary school adolescents are frequently exposed to risky behaviors such as substance use, sexual coercion, and unsafe sex, often compounded by poor parent–child communication, limited access to accurate sexual health information, and weak youth-friendly services [4–6] Although several school- and community-based interventions—such as peer education, anti-AIDS clubs, and youth-friendly reproductive health services—have been implemented in Ethiopia, unsafe sexual practices among adolescents remain prevalent [6]. This suggests that existing interventions may not sufficiently address the behavioral, psychological, and contextual determinants of adolescent sexual behavior.

Risky sexual behaviors among in-school adolescents are influenced by multiple interrelated factors, including developmental changes during adolescence, limited parental supervision and communication, peer pressure, substance use, and misperceptions about sexual risk [2, 6–9]. These factors interact to increase adolescents’ vulnerability to unsafe sexual practices, including unprotected sex and multiple sexual partnerships [9].

Based on the Health Belief Model (HBM), this study aimed to assess unsafe sexual practices by examining how adolescents’ perceptions of susceptibility, severity, benefits, barriers, and self-efficacy—together with socio-demographic and behavioral factors—influence engagement in unsafe sexual behaviors [10]. The findings are expected to inform context-specific interventions and strengthen adolescent sexual and reproductive health programs in the region.

Understanding these individual, social, and psychological determinants is essential for designing effective, theory-based interventions aimed at reducing unsafe sexual practices among adolescents, particularly in under-researched settings such as Gambella town. However, little is known about the concerns in less developed parts of Ethiopia, like the Gambella region. Specifically, information about the level of unsafe sexual practice and factors associated with unsafe sexual practice is limited in Gambella town. Importantly, secondary school adolescents, despite the fact that risk and vulnerability are high in this age group, have research gaps, particularly in the developing regions of Ethiopia, where there is limited information to mitigate the risks. This study is aimed at determining the level of unsafe sexual practices and the factors influencing such activities among adolescents.

## Methods

### Study area, design and period

Institution-based cross-sectional study was conducted in Gambella town among high school students from May 1 to June 20, 2023. Gambella town is located 780 km from the capital city of Ethiopia, Addis Ababa. In the town, there are two secondary schools: Elay Secondary School and Gambella Secondary School. The total number of students in both schools in the 2022/2023 academic year was 3,915 [11].

### Sample size determination

The sample size was determined using the following formula: $\:\mathrm{n}=\frac{{z}^{2}\left(1-p\right)p}{{d}^{2}}$ = 377, p was taken from a study conducted in Jimma zone, Ethiopia [9], which was 57% Z-value of 1.96 is at 95% CI, p: proportion of unsafe sexual practice from previous studies, and d: the margin of error is 5% with an expected non-response rate of 10% and a design effect of 1.5. The sample was calculated for each specific objective, and the higher sample was taken. The sample size for the second objective was calculated by considering different factors associated with unsafe sexual practice using the using the STATCALC of Epi info 7 for different variables with the following assumptions: two-sided confidence level: 95%, margin of error: 5%, ratio: 1:1 (assuming equal group sizes), and power: 80%, and the highest among these resulted in sample size of 270 subjects. Sample size of the first objective was higher (621), and was chosen and finally corrected for finite population.

### Eligibility criteria

Both male and female adoleoecent (aged 10–19 years) students enrolled in selected highschools who provide assent, and whose parents provide informed consent (if under 18)and present during data collection period were included in the study. Students who were absent during data collection or unable to complete the questionnaire due to illness were excluded.

### Sampling technique

**Fig. 1:**
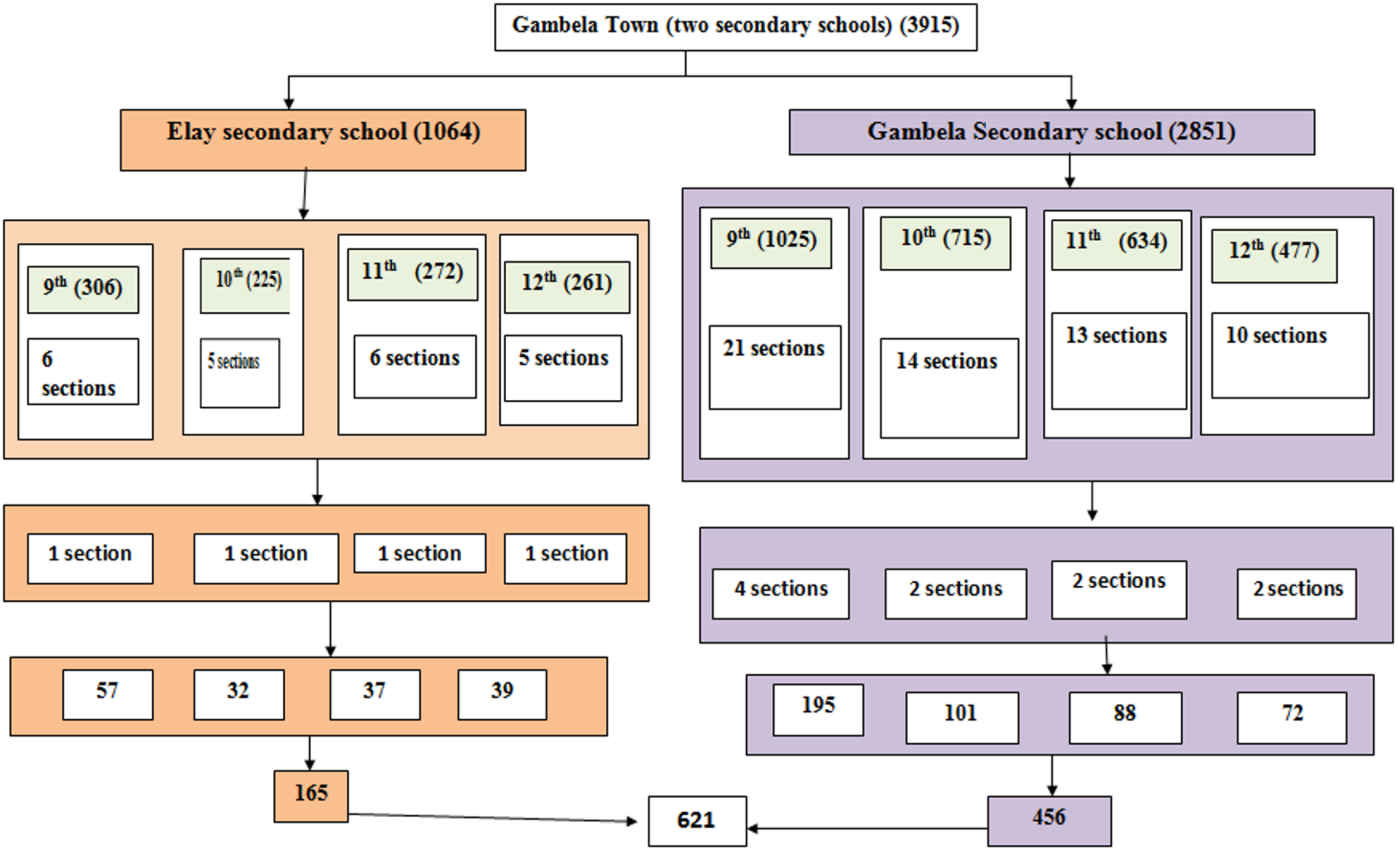
Schematic presentation of sampling procedures to assess unsafe sexual practice, and associated factors among adolescents in secondary schools, Gambella town, Ethiopia, 2023

Stratified sampling was first used to stratify participants by school and grade level to ensure representativeness. Simple random sampling was then applied within each stratum to select study participants. There are two secondary schools (grade 9th -12th ) in the town. Based on the number of sections in each grade (9-12th ), representative number of sections were determined. Then, sample size was proportionally allocated to each section based on the number of students. Finally, study participants were selected by simple random sampling technique using student registration as a sampling frame (Fig. 1).

### Study variables

Unsafe sexual practices were the outcome variable in this study. Independent variables were age, sex, educational status, family status, personality, peer pressure, substance abuse, perceived susceptibility to HIV/AIDS, perceived severity of HIV/AIDS, perceived benefit of safe sexual practice, perceived barriers to abstinence from sexual intercourse and condom use, and perceived self-efficacy for abstinence and condom use.

### Operational definition

Unsafe sexual practice: presence of atleast one of the following: ever having sexual intercourse (premarital) and sexual practice during the last 12 months, having multiple sexual partners during the last 12 months or unprotected sex/not using condoms during sexual intercourse in the past 12 months.

### Data collection tools, procedures and quality assurance

Data were collected using a self-administered questionnaire adapted from previously validated instruments [2, 6, 12–19]. Content validity was assessed by public health and reproductive health experts. The questionnaire was written in English first, then translated into Amharic, and then back to English to ensure consistency. The assessment mainly depended on the primary data collection method using a structured questionnaire. All secondary school students who were attending their education in Gambella town in this academic year (2022–2023)and who were in the class during data collection, were able to give informed consent, and were willing to participate in the study were included. The data collection tool was pre-tested in nearby schools outside of the study area with 31 students (5% of the sample size). The questionnaire was tested for clarity, acceptability, flow, and repetition in a similar setting, and appropriate modifications were made to the final version. The final version of the questionnaire was used for data collection. Structured self-administered questionnaires consisting of sociodemographics, risky sexual behavior, and unsafe sex practices were collected from each participant. The data collection was completed within two days to avoid contamination of the information. Before starting the questionnaire’s distribution, the respondents were briefed about the purpose of the study after getting their informed consent. A minimum of one facilitator who can speak the local language facilitates the data collection process per classroom. A total of four facilitators were selected; those facilitators, who are degree-holder nurses, were selected randomly from nearby health centers. Potential social desirability bias was minimized through anonymity and non-teacher data collectors. Reliability was assessed using appropriate statistical measures (Cronbach’s alpha).

Perceived susceptibility to HIV/AIDS was assessed using multiple questions containing nine questions with yes/no answers. The value above the median was considered “yes”, and the value below the median was considered “no”. Similarly, the perceived severity of HIV/AIDS was measured using two questions and compiled, considering the computed variable, taking the media value and the value above the median value as yes and below the value as no. The perceived benefit of safe sexual practice was assessed using three questions using yes/no answers and computed with the median value, with values above the median as good and below the median as poor.

Perceived barriers to practicing safer behavior were measured using scale measurements ranging from 1 to 5, where the value 1 indicates strongly disagreeing and the value 5 indicates strongly agreeing. The perceived barrier to practicing safer behavior was computed considering the mean value of the seven questions. The value was recategorized as high and low (i.e., above the mean high and below the mean low).

Perceived self-efficacy for abstinence was measured using scale measurements ranging from 1 to 5, with a lower value of 1 for strongly disagreeing and the largest value of 5 for strongly agreeing. Perceived self-efficacy for condom use was measured using scale measurements ranging from 1 to 5, with a lower value of 1 for strongly disagreeing and the largest value of 5 for strongly agreeing. The perceived self-efficacy for practicing safer behavior was computed considering the mean value of the three questions. The value was recategorized as high and low (i.e., above the mean high and below the mean low).

### Data processing and analysis

The collected data was coded, entered in EpiData software, and analysed using SPSS version 25. Descriptive statistics were computed to determine the magnitude of unsafe sexual practices and the factors associated with unsafe sexual practices. A univariate analysis was conducted to assess the association between dependent variables and independent variables with a cut point of a p-value less than 0.25, and variables below the cut point were analysed with a multivariate binary logistic regression analysis in the final model. The variables with a p-value less than 0.05 were reported as significant factors. Odds ratios and 95% confidence intervals were used to forecast risk probabilities.

## Results

### Socio-demographic characteristics

A total of 621 students participated, with a response rate of 100%. The mean age of the respondents was 18.2 (SD: 1.9) years. The study involved students in grades 9–12, with 541 urban residents and 80 rural residents. The majority lived with their families, with 72.8% living with their families, 16.7% with relatives, and 1.5% in rented houses (Table 1).

**Table 1:** Socio-demographic characteristics and risky behaviors among adolescents in secondary schools, Gambela, Ethiopia, 2023

Variables	Categries	Frequency	Percentages
Age category	15–19 20–24	219 402	35.3 64.7
Sex	Male Female	261 360	42 58
Grade	9th 10th 11th 12th	252 133 125 111	40.6 21.4 20.1 17.9
Residence	Urban Rural	541 80	87.1 12.9
Living Arrengements	With family With relatives Rented house with peers	452 104 65	72.8 16.7 1.5
Father’s occupation	Farmer Day laborer Employed worker Has own business	152 121 200 148	24.5 19.5 32.2 23.8
Mother’s occupation	Housewife Daily laborer Employed worker Has own business	261 82 139 139	42 13.2 22.4 22.4
Watched sex film in the last 12 months	Yes No	231 390	37.2 62.8
Chewed khat in the last 12 months	Yes No	118 503	19 81
Drunk alcohol in the last 12 months	Yes No	145 476	23.3 76.7

### Perceived susceptibility and perceived severity of HIV/AIDS

The majority of participants (92.9%) believed that HIV can be transmitted through sexual intercourse, with 87.9% stating that multiple sexual partners increase the risk of infection. Overall, 81% of participants perceived susceptibility to HIV/AIDS and other sexually transmitted infections. The perceived severity of HIV/AIDS among the participants was: 470 (75.7%) perceived that HIV/AIDS has no cure, so that can lead to death, and 505 (81.3%) perceived that unwanted pregnancy and unsafe abortion can cause the death of women. The overall perceived severity was 412 (66.3%) (Table 2).

**Table 2:** Perceived susceptibility to HIV/AIDS and perceived severity of HIV/AIDS among adolescents in secondary schools, Gambella Town, Ethiopia, 2023

Variable		Response	Frequancy(%)
Perceived susceptibility	HIV can be transmitted through sexual intercourse.	Yes	577 (92.9)
		No	44 (7.1)
	Having sexual intercourse with multiple sexual partners can increase exposure to risk of getting HIV infection.	Yes	546 (87.9)
		No	75 (12.1)
	Percieved benefits of abstinence as safer sexual practice	Yes	501 (80.7)
		No	120 (19.3)
	Percieved benefits of condom use as safer sexual practice	Yes	536 (86.3)
		No	85 (13.7)
	Percieved benefits of limiting sexual partners (being in mutual monogamy) as safer sexual practice	Yes	435 (70)
		No	186 (30)
	A healthy looking person can have HIV infections in his/her blood.	Yes	423 (68.1)
		No	198 (31.9)
	If you know a person very well and you trust him/her, you do not have to use condoms when you have sex with him/her.	Yes	226 (36.4)
		No	395 (63.6)
	People who are in monogamous relation are not at risk for HIV infection, so that, they do not need to use condoms.	Yes	249 (40.1)
		No	372 (59.9)
	Over all perceived susceptibility to HIV/AIDS	High	503 (81)
		Low	118 (19)
Perceived severity of sexual health risk	HIV AIDS has no cure, so that can lead to death	Yes	470 (75.7)
		No	151(24.3)
	Unwanted pregnancy and unsafe abortion can cause death of women	Yes	505 (81.3)
		No	116 (18.7)
	Over all perceived severity of HIV/AIDS	High	412 (66.3)
		Low	209 (33.7)

### Perceived benefit of safe sexual practice (abstinence/ using condom)

Majority of respondents, 510 (82.1%) perceived abstinence as one of the most effective methods of avoiding the risk of STIs and HIV/AIDS; 515 (82.9%) perceived consistent condom use as effectively preventing both the risks of STIs and unwanted pregnancy; and about half (49.5%) perceived dual methods as a dual protection option against HIV and unwanted pregnancy. The overall perceived benefit of safe sexual practice among the respondents was 546 (87.9%).

### Perceived social norms and attitude towards safer behavior (abstinence/ using condom)

Regarding barriers to abstinence, only 32.7% of students agreed that peer pressure influences abstaining from sex. Similarly, 22.5% agreed that premarital sex is not against societal or religious norms, and 25% perceived premarital sex as a sign of maturity. For condom use, 35.1% agreed that condoms reduce sexual pleasure, and 28.5% believed condoms are ineffective in preventing pregnancy or STIs. Regarding partner communication, 35.5% agreed that condom use signals distrust, and 32.5% believed requesting condom use could cause conflict. Overall, perceived norms toward abstinence were low for 60.2% of respondents and high for 39.8%. Attitudes toward consistent condom use were low for 47.2% and high for 52.8%, while perceived norms for consistent condom use were low for 56.7% and high for 43.3% (Table 3).

**Table 3:** Attitude and perceived social norms about safer sexual practice of abstinence and condom use among adolescents attending secondary schools, in Gambella Town, Ethiopia, 2023

Variable		Categories	Frequancy	Percentage	Mean value	SD	Overall	
							Low	High
*Perceived norms towards unsafe sexual practice*	Perceived peer norms about premarital sex	SDA*	95	15.3	2.84	1.37	374 (60.2%)	247 (39.8%)
		DA	234	37.7				
		N	89	14.3				
		A	83	13.4				
		SA	120	19.3				
	Premarital sex is acceptable in the society/religion	SDA	102	16.4	2.57	1.23		
		DA	276	44.4				
		N	103	16.6				
		A	66	10.6				
		SA	74	11.9				
	Premarital sex is a sign of maturity	SDA	108	17.4	2.61	1.26		
		DA	261	42.0				
		N	97	15.6				
		A	77	12.4				
		SA	78	12.6				
*Attitude towards consistent use of condom*	Condom use reduces sexual pleasure	SDA	68	11	3.12	1.21	293 (47.2%)	328 (52.8%)
		DA	113	18.2				
		N	222	35.7				
		A	113	18.2				
		SA	105	16.9				
	Condoms are not effective in preventing pregnancy and STIs	SDA	100	16.1	2.65	1.24		
		DA	260	41.9				
		N	84	13.5				
		A	112	18.0				
		SA	65	10.5				
*Perceived norms to consistant condome use*	Condom use and perceived mistrust	SDA	72	11.6	3.03	1.25	352 (56.7%)	269 (43.3%)
		DA	188	30.3				
		N	141	22.7				
		A	127	20.5				
		SA	93	15				
	Partner suspicion towards condom use request	SDA	81	13	3.06	1.26		
		DA	175	28.5				
		N	163	26.2				
		A	104	16.7				
		SA	98	15.8				

Key: *SDA: Strongly disagree, DA: Disagree, N: Neutral, A: Agree, and SA: Strongly agree

### Perceived self-efficacy to avoid unsafe sexual practice

More than half (55.4%)of the respondents demonstrated high perceived self-efficacy for abstaining from sexual intercourse. More than half (52.2%) of participants strongly agreed that they were confident in refusing sexual intercourse when pressured by friends, while 242 (39.0%) strongly agreed that they could effectively say no to sex in situations challenging abstinence. Perceived self-efficacy for condom use was comparatively lower. Among sexually active participants, 198 (31.9%) strongly agreed that they could negotiate condom use with a regular partner or refuse sex if their partner declined to use condoms. Similarly, 191 (31.1%) strongly agreed that they could convince a steady partner to consistently use condoms, and 189 (30.4%) strongly agreed that they could always use condoms even when another contraceptive method was being used. Overall, high perceived self-efficacy for condom use was reported by 283 (45.6%) of sexually active respondents (Table 4).

**Table 4:** Perceived self-efficacy for abstinence and condom use to avoid unsafe sexual practice among adolescents attending secondary schools, in Gambella Town, Ethiopia, 2023

Variable		Category	Frequancy	Percentage	Mean value	SD	Overall	
							**Low**	**High**
*Perceived self-efficacy for abstinence*	refusal of sexual intercourse	SDA	95	15.3	3.8	1.49	277 (44.6%)	344 (55.4%)
		DA	32	5.2				
		N	98	15.8				
		A	72	11.6				
		SA	324	52.2				
	Abstinence from sexual intercourse	SDA	69	11.1	3.58	1.38		
		DA	67	10.8				
		N	163	26.2				
		A	80	12.9				
		SA	242	39				
*Perceived self-efficacy for condom use*	Ability of negotiation for condom use	SDA	77	12.4	3.41	1.35	388 (54.4%)	283 (45.6%)
		DA	66	10.6				
		N	200	32.2				
		A	80	12.9				
		SA	198	31.9				
	Partener persuasion for condom use	SDA	71	11.4	3.51	1.32		
		DA	59	9.5				
		N	168	27.1				
		A	130	20.9				
		SA	193	31.1				
	Consistent condom use	SDA	95	15.3	3.30	1.42		
		DA	82	13.2				
		N	177	28.5				
		A	78	12.6				
		SA	189	30.4				

Key: SDA: Strongly disagree, DA: Disagree, N: Neutral, A: Agree, and SA: Strongly agree

### Unsafe sexual practice

Of the total respondents, 251 (40.4%) reported ever having sexual intercourse. The median age at sexual debut was 16 years. Among sexually experienced students, 105 (41.8%) reported regretting their first sexual encounter, while 154 (61.4%) initiated sexual intercourse based on their own decision and 97 (38.6%) due to external influences. Condom use at first sexual intercourse was reported by only 140 (55.5%) respondents. Regarding recent sexual activity, 227 (90.4%) of those who had ever had sex reported sexual intercourse within the preceding 12 months. Among these, 110 (48.5%) reported inconsistent condom use, 124 (54.6%) had one sexual partner, and 103 (45.4%) reported having two or more sexual partners. Overall, 227 (36.6%) of the respondents engaged in unsafe sexual practices [95% CI: 32.8%–40.4%] (Table 5).

**Table 5:** Unsafe sexual practice among adolescents attending secondary schools, in Gambella Town, Ethiopia, 2023

Variables	Categories	Frequancy	Percentages
Ever had sexual intercourse	Yes No	251 370	40.4 59.6
Own decision to have sex for the first time (*n* = 251)	Yes No	154 97	61.4 38.6
Reason to have sexual intercourse in the first sexual contact (*n* = 97)	Alcohol use Kchat chewing Economic reason Peer presure Partner reinforcement	27 27 18 16 9	27.8 27.8 18.6 16.5 9.3
Condom used during this first time you had sexual intercourse (*n* = 251)	Yes No	140 111	55.8 44.2
Had sexual intercourse in the last 12 months (*n* = 251)	Yes No	227 24	90.4 9.6
Number of sexual partners in last 12 months (*n* = 227)	Only one Two or more	124 103	54.6 45.4
Condom use in the last time you had sexual intercourse* (*n* = 227)	Yes No	110 117	48.5 51.5
Unsafe sexual practice** (*n* = 621)	Yes No	227 394	36.6 63.4

Key: * The frequency of condom use was’sometimes’ for all of them (110) who reported using condoms

**If ever had sexual intercourse and had sexual intercourse in the last 12 months and condom unprotected sex, then Yes; otherwise, No

### Factors associated with unsafe sexual practice

Bivariable logistic regression was performd to identify factors associated with unsafe sexual practice among adolescents. In this analysis, sex, residence, age category, perceived susceptibility, perceived severity of HIV/AIDS, perceived benefit of safe sexual practice, attitude toward consistent condom use, perceived norms of abstinence, perceived norms of consistent condom use, perceived self-efficacy for condom use, watching sex films, chewing khat, and drinking alcohol were selected as candidate variables for unsafe sexual practice at a p-value less than 0.25.

In the multivariable logistic regression analysis, eight [8] variables were significant at a p-value less than 0.05. Accordingly, sex [AOR: 2.38, 95% C.I.: 1.61–3.51], age category [AOR: 0.65, 95% C.I.: 0.43–0.97], perceived norms to abstinence [AOR: 0.60, 95% C.I.: 0.41–0.89], perceived norms to consistent condom use [AOR: 2.05, 95% C.I.: 1.39–3.03], perceived self-efficacy for condom use [AOR: 0.59, 95% C.I.: 0.40–0.86], watching sex films [AOR: 1.50, 95% C.I.: 1.006-2.20], chewing khat [AOR: 2.53, 95% C.I.: 1.57–4.07], and drinking alcohol [AOR: 2.93, 95% C.I.: 1.86–4.62] were significantly associated with unsafe sexual practice.

This finding shows that male students were more than two times more likely to have unsafe sexual practice than female students [AOR: 2.38, 95% C.I: 1.61–3.51], student age 18 years or above were 35% less likely to practice unsafe sexual practice than the age less than 18 [AOR: 0.65, 95% C.I: 0.43–0.97], students those watch sex film were1.5 more likely to practice unsafe sexual practice than their counter part [AOR: 1.50, 95% C.I: 1.006-2.20], students those chew khat were 2.5 times more likely practice unsafe sexual practice than their counter part [AOR: 2.53, 95% C.I: 1.57–4.07], and student those drink alcohol were 2.9 times more likely practice unsafe sexual practice than their counter part [AOR: 2.93, 95% C.I: 1.86–4.62]. Students who perceived low norms to abstinence were 40% less likely [AOR: 0.60, 95% C.I.: 0.41–0.89] to have unsafe sexual practices than those who perceived high norms to abstinence. Students who perceived low norms for consistent condom use were two times more likely [AOR: 2.05, 95% C.I.: 1.39–3.03] to practise unsafe sexual practices than those who perceived high norms for consistent condom use. Similarly, students who perceived low self-efficacy for condom use were 39% less likely [AOR: 0.59, 95% C.I.: 0.40–0.86] to practise unsafe sexual practices than their counterparts (Table 6).

**Table 6:** Multivariable analysis factors associated with unsafe sexual practices among adolescents attending secondary schools in Gambela Town, Ethiopia, 2023

Varaibles	Categories	Unsafe sexual practice		Crude OR	Adjusted OR	
		Yes	No	COR [95% C.I]	AOR [95% C.I]	P-Value
Sex	Male	129	132	2.61 [1.87–3.65]	2.38 [1.61–3.51]	< 0.001*
	Female	98	262	1	1	
Residence	Urban	192	349	0.70 [0.44–1.138]	0.95 [0.55–1.65]	0.868
	Rural	35	45	1	1	
Age category	Less than 18	58	161	0.49 [0.34–0.712]	0.65 [0.43–0.97]	0.038*
	18 and above	169	233	1	1	
Percieved Susseptablity	High	194	309	1.62 [1.04–2.51]	1.49 [0.90–2.47]	0.120
	Low	33	85	1	1	
Percieved severity of HIV/AIDs	High	141	271	0.74 [0.53–1.05]	1.04 [0.70–1.56]	0.822
	Low	86	123	1	1	
Percieved Benefit of safe sexual practice	High	205	341	1.45 [0.856–2.45]	1.54 [0.86–2.79]	0.146
	Low	22	53	1	1	
Perceived norms to abstainance	Low	115	259	0.53 [0.38–0.74]	0.60 [0.41–0.89]	0.013*
	High	112	135	1	1	
Attitude to concestant condome use	Low	99	194	0.79 [0.57–1.07]	1.17 [0.79–1.72]	0.436
	High	128	200	1	1	
Perceived norms to concestant condome use	Low	149	203	1.79 [1.28–2.52]	2.05 [1.39–3.03]	< 0.001*
	High	78	191	1	1	
Perceived self-efficacy for condom use	Low	109	229	0.66 [0.48–0.92]	0.59 [0.40–0.86]	0.007*
	High	118	165	1	1	
Watch sex film	Yes	114	117	2.39 [1.70–3.35]	1.50 [1.01–2.20]	0.047*
	No	113	277	1	1	
Chew khat	Yes	69	49	3.07 [2.04–4.64]	2.53 [1.57–4.07]	< 0.001*
	No	158	345	1	1	
Drink alcohol	Yes	83	62	3.09 [2.105–4.53]	2.93 [1.86–4.62]	< 0.001*
	No	144	332	1	1	

Key: *siginficant at p-value less than 0.05

## Discussion

This school-based study assessed the magnitude of unsafe sexual practice and its associated factors among secondary school students using constructs of the Health Belief Model (HBM). The findings indicate that unsafe sexual practice remains a substantial public health concern among adolescents in the study setting.

In this study, 40.4% of students reported ever having sexual intercourse, with a median age at sexual debut of 16 years. This finding is consistent with previous studies conducted in Ethiopia and Nigeria, which report early initiation of sexual activity among adolescents [6,25–27]. Early sexual debut exposes adolescents to a higher risk of unsafe sexual practices, unintended pregnancy, and sexually transmitted infections (STIs), including HIV.

More than one-third (36.6%) of respondents engaged in unsafe sexual practices. This prevalence is comparable with pooled estimates from a national meta-analysis in Ethiopia [20], but higher than reports from Nigeria [21], Arba Minch town in southern Ethiopia [13], and Aksum town in northern Ethiopia [22]. These discrepancies may be attributed to differences in study period, sociocultural context, and the distribution of behavioural risk factors across settings.

Sociodemographic factors were significantly associated with unsafe sexual practice. Male students were more than twice as likely to engage in unsafe sexual practices compared to female students. This finding aligns with evidence from a systematic review in Ethiopia showing higher risk-taking behavior among male youths [20]. Gender norms that encourage sexual experimentation among males while discouraging similar behavior among females may partly explain this difference.

Age was also a significant predictor; students aged 18 years or younger were less likely to practice unsafe sexual behaviors compared to older students. This finding is consistent with studies from South Africa, where older adolescents were more likely to engage in risky sexual practices [23]. Increased autonomy, exposure to substances, and peer influence among older adolescents may contribute to this pattern.

Behavioral factors, including watching pornography, alcohol consumption, and khat chewing, were strongly associated with unsafe sexual practice. Students who reported watching pornographic films were more likely to engage in unsafe sexual practices, a finding consistent with pooled evidence from Ethiopia and studies conducted among high school students in Gondar [6, 24]. Exposure to sexual content may normalize risky behaviors and reduce perceived susceptibility to adverse sexual health outcomes.

Alcohol consumption was one of the strongest predictors of unsafe sexual practice. Students who consumed alcohol were nearly three times more likely to engage in unsafe sexual behaviors, consistent with findings from Gondar, Bahir Dar, and Kigali, Rwanda [17, 24, 25]. Alcohol may impair judgment and decision-making capacity, leading to unprotected intercourse or unintended sexual encounters.

Similarly, khat chewing was significantly associated with unsafe sexual practice, which is supported by studies from Alamata, Dessie town, and Dessie Zuria woreda [14, 18]. Risk behaviors often cluster together, and khat use may increase susceptibility to other substances, such as alcohol, thereby compounding sexual risk-taking behaviors [26].

HBM-related perceptions were also significantly associated with unsafe sexual practice. Students who perceived low social norms supporting consistent condom use were more likely to engage in unsafe sexual behaviors. Likewise, perceived self-efficacy for condom use showed a significant association, consistent with findings from western Nigeria [21]. These findings underscore the importance of enhancing adolescents’ confidence and social support for consistent condom use.

Perceived norms regarding abstinence were also associated with unsafe sexual practice. Students who perceived abstinence as socially acceptable and positive were less likely to engage in unsafe sexual behaviors. In this study, low perceived norms for abstinence—defined by beliefs that premarital sex is common, socially acceptable, or a sign of maturity—were protective against unsafe sexual practice. This suggests that adolescents who critically perceive peer norms may resist pressure to engage in risky sexual behaviors. Similar findings have been reported elsewhere, highlighting the role of perceived norms in shaping abstinence behaviors [27].

### Strengths and limitations

The cross-sectional design limits the ability to establish causal relationships. Data were collected using self-administered questionnaires, which are subject to recall and social desirability bias, particularly for sensitive behaviors such as sexual practices and substance use. Additionally, the absence of interviewer guidance and the inability to apply complex skip patterns may have resulted in missing or misreported information.

Despite these limitations, the study has notable strengths. A large sample size and random sampling technique were used to enhance representativeness. Rigorous data quality control measures were implemented to minimize bias and improve the reliability of the findings. The use of a theory-based framework (HBM) also strengthens the interpretation of behavioral and perceptual factors associated with unsafe sexual practice.

## Conclusions

The study found that about two in five students in the setting engaged in unsafe sexual practices. Factors such as sex, age, perceived norms to abstain, constant condom use, self-efficacy for condom use, watching sex films, chewing khat, and drinking alcohol were significantly associated with unsafe sexual practices among adolescents in the study setting. The Gembella town education office, health office, and school communities are recommended to play their role in reducing behavioral factors around the school environment, in-school health education targeted to their age and gender, and reducing the vulnerability of school youths to unsafe sexual practices. Additionally, intensive interventional research is also important to mitigate the consequences of unsafe sexual practices among in-school youths.

## Supplementary Information

Below is the link to the electronic supplementary material.

Supplementary Material 1.

Supplementary Material 2.

## Data Availability

The datasets used during the current study are available and can be accessed from the corresponding author upon reasonable request.

## Abbreviations

AIDS: Acquired Immunodefiency Syndrome
AOR: Adjusted Odds Ratio
CI: Confidence Interval
COR: Crude Odds Ratio
HBM: Health Belief Model
HIV: Human Immunodeficency Virus
STI: Sexually transmitted infection
SPSS: Statistical Package for Social Sciences
USP: unsafe sexual practice
WHO: World Health Organization
