# Supplementary material for: Unsafe sexual practice and associated factors among adolescents in secondary schools in Gambella town, Ethiopia: a health belief model
Source: BMC Public Health. 2026 Apr 22;26:1797. doi: 10.1186/s12889-026-27466-2 (PMC13235138; doi:10.1186/s12889-026-27466-2)
Supplement: Supplementary file 2 — Supplementary Material 2. [file 12889_2026_27466_MOESM2_ESM.docx]

**Unsafe sexual practice and associated factors among adolescents in secondary schools in Gambella town, Ethiopia: a health belief model**

**Tariku Frissa^a^, Asrat Zewdie^a*^, Dereje Oljira Donacho^b^, and Benti Negero^c^**

^a^ Department of Public Health, College of Health Science, Mattu University, Mattu, Ethiopia

^b^ Department of Health Informatics, College of Health Science, Mattu University, Mattu, Ethiopia

^c^ Department of Midwifery, College of Health Science, Mattu University, Mattu, Ethiopia

*Corresponding author: **Asrat Zewdie** **Email:** [**asratzewdie49@gmail.com**](mailto:asratzewdie49@gmail.com)

**^a^[erkofri@gmail.com](mailto:erkofri@gmail.com)**

**^b^[dodbau3687@gmail.com](mailto:dodbau3687@gmail.com)**

**^c^** [**nagaro_b@yahoo.com**](mailto:nagaro_b@yahoo.com)

# Abstract

**Background:** Unsafe sexual practices among adolescents represent a significant public health challenge due to their association with increased risk of sexually transmitted infections, including HIV, and unintended pregnancies. The burden remains substantial, contributing to adverse health outcomes, educational disruption and long-term socio-economic consequences. Multiple factors like inadequate sexual health knowledge, limited access to youth-friendly services, low utilisation of preventive methods, and social and behavioural determinants such as peer pressure, substance use, early sexual initiation and weak parental communication further exacerbate the problem.

**Methods:** An institution-based cross-sectional survey using a self-administered questionnaire was administered to 621 students who were selected using the systematic sampling technique. Unsafe sexual practice was measured using the self-reported behavioural indicators, including inconsistent condom use, multiple sexual partners, and early sexual initiation. The Health Belief Model (HBM) was used to assess psychosocial determinants of these behaviours. The HBM constructs (perceived susceptibility, perceived severity, perceived benefits, perceived barriers, self-efficacy, and cues to action) were measured using validated Likert-scale items. Composite scores were generated for each construct and analyzed to determine their association with unsafe sexual practices. Corresponding p-values and adjusted odds ratios with 95% confidence intervals have been added to improve clarity and statistical interpretation.

**Results:** The prevalence of unsafe sexual practices was 36.6% [95% CI: 32.8%–40.4%]. The study found that male sex (AOR= 2.38; 95% C.I.: 1.61-3.51, p<0.001), age less than 18 years (AOR= 0.65, 95% C.I.: 0.43-0.97, p=0.038), low perceived norms for abstinence [AOR= 0.6, 95% C.I.: 0.41-0.89, p=0.013], low perceived norms for consistent condom use [AOR= 2.05, 95% C.I.: 1.39-3.03, p<0.001], low perceived self-efficacy for condom use [AOR= 0.59, 95% C.I.: 0.40-0.86, p=0.007], watching sex films [AOR= 1.5, 95% C.I.: 1.01-2.20, p=0.047], chewing khat [AOR= 2.5, 95% C.I.: 1.57-4.07, p<0.001], and drinking alcohol [AOR= 2.93, 95% C.I: 1.86-4.62, p<0.001] were significantly associated with unsafe sexual practice among adolescents.

**Conclusion:** The study revealed that about two in five students engage in unsafe sexual practices, influenced by socio-demographic and behavioural factors, highlighting the need for collaboration between key stakeholders to mitigate risks and consequences.

***Key words:*** *Unsafe sexual practice, secondary sc**hool, Gambela, Health Belif model*

# Background

Adolescence, defined by the World Health Organization as the period between 10 and 19 years of age, is a critical developmental stage characterized by rapid physical, psychological, and social changes that may predispose young people to risky behaviors(1). Unsafe sexual practices—such as early sexual debut, inconsistent or non-use of condoms, multiple sexual partners, and engagement with high-risk partners—remain a major public health concern among adolescents, particularly in developing countries (1,2). These behaviors substantially increase the risk of sexually transmitted infections (STIs), including HIV/AIDS, unintended pregnancy, unsafe abortion, and their associated social, educational, and economic consequences (2).

Globally, adolescents constitute about 20% of the population, with nearly 90% living in low- and middle-income countries (3). Sub-Saharan Africa bears a disproportionate share of the burden of adverse sexual and reproductive health outcomes among adolescents, including high rates of HIV infection, early pregnancy, and school dropout(4,5) . In this region, adolescents and youths account for approximately 20–30% of the total population, making unsafe sexual practices a significant public health and development challenge (5). Secondary school students, in particular, are vulnerable due to experimentation, peer pressure, limited life experience, and a perceived sense of invulnerability (5).

Evidence from African countries shows that secondary school adolescents are frequently exposed to risky behaviors such as substance use, sexual coercion, and unsafe sex, often compounded by poor parent–child communication, limited access to accurate sexual health information, and weak youth-friendly services (4–6) Although several school- and community-based interventions—such as peer education, anti-AIDS clubs, and youth-friendly reproductive health services—have been implemented in Ethiopia, unsafe sexual practices among adolescents remain prevalent (6). This suggests that existing interventions may not sufficiently address the behavioral, psychological, and contextual determinants of adolescent sexual behavior.

Risky sexual behaviors among in-school adolescents are influenced by multiple interrelated factors, including developmental changes during adolescence, limited parental supervision and communication, peer pressure, substance use, and misperceptions about sexual risk(2,6–9). These factors interact to increase adolescents’ vulnerability to unsafe sexual practices, including unprotected sex and multiple sexual partnerships (9).

Based on the Health Belief Model (HBM), this study aimed to assess unsafe sexual practices by examining how adolescents’ perceptions of susceptibility, severity, benefits, barriers, and self-efficacy—together with socio-demographic and behavioral factors—influence engagement in unsafe sexual behaviors (10). The findings are expected to inform context-specific interventions and strengthen adolescent sexual and reproductive health programs in the region.

Understanding these individual, social, and psychological determinants is essential for designing effective, theory-based interventions aimed at reducing unsafe sexual practices among adolescents, particularly in under-researched settings such as Gambella town. However, little is known about the concerns in less developed parts of Ethiopia, like the Gambella region. This study's findings are crucial for evaluating the level of unsafe sexual practices and the factors influencing such activities. Specifically, information about the level of unsafe sexual practice and factors associated with unsafe sexual practice is limited in Gambella town. Importantly, secondary school adolescents, despite the fact that risk and vulnerability are high in this age group, have research gaps, particularly in the developing regions of Ethiopia, where there is limited information to mitigate the risks.

# Methods

## Study area, design and period

Institution-based cross-sectional study was conducted in Gambella town among high school students from May 1 to June 20, 2023. Gambella town is located 780 km from the capital city of Ethiopia, Addis Ababa. In the town, there are two secondary schools: Elay Secondary School and Gambella Secondary School. The total number of students in both schools in the 2022/2023 academic year was 3,915 (11).

## Sample size determination

The sample size was determined using the following formula: $n=\frac{z^{2}\left( 1-p \right)p}{d^{2}}$ = 377, p was taken from a study conducted in Jimma zone, Ethiopia, (9) which was 57% Z-value of 1.96 is at 95% CI, p: proportion of unsafe sexual practice from previous studies, and d: the margin of error is 5% with an expected non-response rate of 10% and a design effect of 1.5. The sample was calculated for each specific objective, and the higher sample was taken. The sample size for the second objective was calculated by considering different factors associated with unsafe sexual practice using the using the STATCALC of Epi info 7 for different variables with the following assumptions: two-sided confidence level: 95%, margin of error: 5%, ratio: 1:1 (assuming equal group sizes), and power: 80%, and the highest among these resulted in sample size of 270 subjects. Sample size of the first objective was higher (621), and was chosen and finally corrected for finite population.

## Eligibility criteria

Both male and female adoleoecent (aged 10-19 years) students enrolled in selected highschools who provide assent, and whose parents provide informed consent (if under 18)and present during data collection period were included in the study. Students who were absent during data collection or unable to complete the questionnaire due to illness were excluded.

## Sampling technique

Stratified sampling was first used to stratify participants by school and grade level to ensure representativeness. Simple random sampling was then applied within each stratum to select study participants. There are two secondary schools (grade 9^th^ -12^th^) in the town. Based on the number of sections in each grade (9-12^th^ ), representative number of sections were determined. Then, sample size was proportionally allocated to each section based on the number of students. Finally, study participants were selected by simple random sampling technique using student registration as a sampling frame (Figure 1).

## Study variables

Unsafe sexual practices were the outcome variable in this study. Independent variables were age, sex, educational status, family status, personality, peer pressure, substance abuse, perceived susceptibility to HIV/AIDS, perceived severity of HIV/AIDS, perceived benefit of safe sexual practice, perceived barriers to abstinence from sexual intercourse and condom use, and perceived self-efficacy for abstinence and condom use.

## Operational definition

**Unsafe sexual practice: presence of atleast one of the following: ever having sexual intercourse (premarital) and sexual practice during the last 12 months, having multiple sexual partners during the last 12 months or unprotected sex/not using condoms during sexual intercourse in the past 12 months.**

## Data collection tools, procedures and quality assurance

Data were collected using a self-administered questionnaire adapted from previously validated instruments (2,6,12–19). Content validity was assessed by public health and reproductive health experts. The questionnaire was written in English first, then translated into Amharic, and then back to English to ensure consistency. The assessment mainly depended on the primary data collection method using a structured questionnaire. All secondary school students who were attending their education in Gambella town in this academic year (2022-2023)and who were in the class during data collection, were able to give informed consent, and were willing to participate in the study were included. The data collection tool was pre-tested in nearby schools outside of the study area with 31 students (5% of the sample size). The questionnaire was tested for clarity, acceptability, flow, and repetition in a similar setting, and appropriate modifications were made to the final version. The final version of the questionnaire was used for data collection. Structured self-administered questionnaires consisting of sociodemographics, risky sexual behavior, and unsafe sex practices were collected from each participant. The data collection was completed within two days to avoid contamination of the information. Before starting the questionnaire's distribution, the respondents were briefed about the purpose of the study after getting their informed consent. A minimum of one facilitator who can speak the local language facilitates the data collection process per classroom. A total of four facilitators were selected; those facilitators, who are degree-holder nurses, were selected randomly from nearby health centers. Potential social desirability bias was minimized through anonymity and non-teacher data collectors. Reliability was assessed using appropriate statistical measures (Cronbach’s alpha).

Perceived susceptibility to HIV/AIDS was assessed using multiple questions containing nine questions with yes/no answers. The value above the median was considered “yes”, and the value below the median was considered “no”. Similarly, the perceived severity of HIV/AIDS was measured using two questions and compiled, considering the computed variable, taking the media value and the value above the median value as yes and below the value as no. The perceived benefit of safe sexual practice was assessed using three questions using yes/no answers and computed with the median value, with values above the median as good and below the median as poor.

Perceived barriers to practicing safer behavior were measured using scale measurements ranging from 1 to 5, where the value 1 indicates strongly disagreeing and the value 5 indicates strongly agreeing. The perceived barrier to practicing safer behavior was computed considering the mean value of the seven questions. The value was recategorized as high and low (i.e., above the mean high and below the mean low).

Perceived self-efficacy for abstinence was measured using scale measurements ranging from 1 to 5, with a lower value of 1 for strongly disagreeing and the largest value of 5 for strongly agreeing. Perceived self-efficacy for condom use was measured using scale measurements ranging from 1 to 5, with a lower value of 1 for strongly disagreeing and the largest value of 5 for strongly agreeing. The perceived self-efficacy for practicing safer behavior was computed considering the mean value of the three questions. The value was recategorized as high and low (i.e., above the mean high and below the mean low).

## Data processing and analysis

The collected data was coded, entered in EpiData software, and analysed using SPSS version 25. Descriptive statistics were computed to determine the magnitude of unsafe sexual practices and the factors associated with unsafe sexual practices. A univariate analysis was conducted to assess the association between dependent variables and independent variables with a cut point of a p-value less than 0.25, and variables below the cut point were analysed with a multivariate binary logistic regression analysis in the final model. The variables with a p-value less than 0.05 were reported as significant factors. Odds ratios and 95% confidence intervals were used to forecast risk probabilities.

## Results

## Socio-demographic characteristics

A total of 621 students participated, with a response rate of 100%. The mean age of the respondents was 18.2 (SD: 1.9) years. The study involved students in grades 9–12, with 541 urban residents and 80 rural residents. The majority lived with their families, with 72.8% living with their families, 16.7% with relatives, and 1.5% in rented houses ([Table 1](#_Table_1:_Socio-demographic)).

## Perceived susceptibility and perceived severity of HIV/AIDS

The majority of participants (92.9%) believed that HIV can be transmitted through sexual intercourse, with 87.9% stating that multiple sexual partners increase the risk of infection. Overall, 81% of participants perceived susceptibility to HIV/AIDS and other sexually transmitted infections. The perceived severity of HIV/AIDS among the participants was: 470 (75.7%) perceived that HIV/AIDS has no cure, so that can lead to death, and 505 (81.3%) perceived that unwanted pregnancy and unsafe abortion can cause the death of women. The overall perceived severity was 412 (66.3%) ([Table 2](#_Table_2:_Perceived)).

## Perceived benefit of safe sexual practice (abstinence/ using condom)

Majority of respondents, 510 (82.1%) perceived abstinence as one of the most effective methods of avoiding the risk of STIs and HIV/AIDS; 515 (82.9%) perceived consistent condom use as effectively preventing both the risks of STIs and unwanted pregnancy; and about half (49.5%) perceived dual methods as a dual protection option against HIV and unwanted pregnancy. The overall perceived benefit of safe sexual practice among the respondents was 546 (87.9%)

## Perceived social norms and attitude towards safer behavior (abstinence/ using condom)

Regarding barriers to abstinence, only 32.7% of students agreed that peer pressure influences abstaining from sex. Similarly, 22.5% agreed that premarital sex is not against societal or religious norms, and 25% perceived premarital sex as a sign of maturity. For condom use, 35.1% agreed that condoms reduce sexual pleasure, and 28.5% believed condoms are ineffective in preventing pregnancy or STIs. Regarding partner communication, 35.5% agreed that condom use signals distrust, and 32.5% believed requesting condom use could cause conflict. Overall, perceived norms toward abstinence were low for 60.2% of respondents and high for 39.8%. Attitudes toward consistent condom use were low for 47.2% and high for 52.8%, while perceived norms for consistent condom use were low for 56.7% and high for 43.3% ([Table 3](#_Table_3:_Attitude)).

## Perceived self-efficacy to avoid unsafe sexual practice

More than half (55.4%)of the respondents demonstrated high perceived self-efficacy for abstaining from sexual intercourse. More than half (52.2%) of participants strongly agreed that they were confident in refusing sexual intercourse when pressured by friends, while 242 (39.0%) strongly agreed that they could effectively say no to sex in situations challenging abstinence. Perceived self-efficacy for condom use was comparatively lower. Among sexually active participants, 198 (31.9%) strongly agreed that they could negotiate condom use with a regular partner or refuse sex if their partner declined to use condoms. Similarly, 191 (31.1%) strongly agreed that they could convince a steady partner to consistently use condoms, and 189 (30.4%) strongly agreed that they could always use condoms even when another contraceptive method was being used. Overall, high perceived self-efficacy for condom use was reported by 283 (45.6%) of sexually active respondents ([Table 4](#_Table_4:_Perceived)).

## Unsafe sexual practice

Of the total respondents, 251 (40.4%) reported ever having sexual intercourse. The median age at sexual debut was 16 years. Among sexually experienced students, 105 (41.8%) reported regretting their first sexual encounter, while 154 (61.4%) initiated sexual intercourse based on their own decision and 97 (38.6%) due to external influences. Condom use at first sexual intercourse was reported by only 140 (55.5%) respondents. Regarding recent sexual activity, 227 (90.4%) of those who had ever had sex reported sexual intercourse within the preceding 12 months. Among these, 110 (48.5%) reported inconsistent condom use, 124 (54.6%) had one sexual partner, and 103 (45.4%) reported having two or more sexual partners. Overall, 227 (36.6%) of the respondents engaged in unsafe sexual practices [95% CI: 32.8%–40.4%] ([Table 6](#_Table_5:_Unsafe)).

### Factors associated with unsafe sexual practice

Bivariable logistic regression was performd to identify factors associated with unsafe sexual practice among adolescents. In this analysis, sex, residence, age category, perceived susceptibility, perceived severity of HIV/AIDS, perceived benefit of safe sexual practice, attitude toward consistent condom use, perceived norms of abstinence, perceived norms of consistent condom use, perceived self-efficacy for condom use, watching sex films, chewing khat, and drinking alcohol were selected as candidate variables for unsafe sexual practice at a p-value less than 0.25.

In the multivariable logistic regression analysis, eight (8) variables were significant at a p-value less than 0.05. Accordingly, sex [AOR: 2.38, 95% C.I.: 1.61-3.51], age category [AOR: 0.65, 95% C.I.: 0.43-0.97], perceived norms to abstinence [AOR: 0.60, 95% C.I.: 0.41-0.89], perceived norms to consistent condom use [AOR: 2.05, 95% C.I.: 1.39-3.03], perceived self-efficacy for condom use [AOR: 0.59, 95% C.I.: 0.40-0.86], watching sex films [AOR: 1.50, 95% C.I.: 1.006-2.20], chewing khat [AOR: 2.53, 95% C.I.: 1.57-4.07], and drinking alcohol [AOR: 2.93, 95% C.I.: 1.86-4.62] were significantly associated with unsafe sexual practice.

This finding shows that male students were more than two times more likely to have unsafe sexual practice than female students [AOR:  2.38, 95% C.I: 1.61-3.51], student age 18 years or above were 35 % less likely to practice unsafe sexual practice than the age less than 18 [AOR:  0.65, 95% C.I: 0.43-0.97], students those watch sex film were1.5 more likely to practice unsafe sexual practice than their counter part [AOR:  1.50, 95% C.I: 1.006-2.20], students those chew khat were 2.5 times more likely practice unsafe sexual practice than their counter part [AOR:  2.53, 95% C.I: 1.57-4.07], and student those drink alcohol were 2.9 times more likely practice unsafe sexual practice than their counter part [AOR:  2.93, 95% C.I: 1.86-4.62]. Students who perceived low norms to abstinence were 40% less likely [AOR: 0.60, 95% C.I.: 0.41-0.89] to have unsafe sexual practices than those who perceived high norms to abstinence. Students who perceived low norms for consistent condom use were two times more likely [AOR: 2.05, 95% C.I.: 1.39–3.03] to practise unsafe sexual practices than those who perceived high norms for consistent condom use. Similarly, students who perceived low self-efficacy for condom use were 39% less likely [AOR: 0.59, 95% C.I.: 0.40-0.86] to practise unsafe sexual practices than their counterparts ([Table 6](#_Table_6:_Multivariable)).

# Discussion

This school-based study assessed the magnitude of unsafe sexual practice and its associated factors among secondary school students using constructs of the Health Belief Model (HBM). The findings indicate that unsafe sexual practice remains a substantial public health concern among adolescents in the study setting.

In this study, 40.4% of students reported ever having sexual intercourse, with a median age at sexual debut of 16 years. This finding is consistent with previous studies conducted in Ethiopia and Nigeria, which report early initiation of sexual activity among adolescents [6,25–27]. Early sexual debut exposes adolescents to a higher risk of unsafe sexual practices, unintended pregnancy, and sexually transmitted infections (STIs), including HIV.

More than one-third (36.6%) of respondents engaged in unsafe sexual practices. This prevalence is comparable with pooled estimates from a national meta-analysis in Ethiopia (20), but higher than reports from Nigeria (21), Arba Minch town in southern Ethiopia (13), and Aksum town in northern Ethiopia (22). These discrepancies may be attributed to differences in study period, sociocultural context, and the distribution of behavioural risk factors across settings.

Sociodemographic factors were significantly associated with unsafe sexual practice. Male students were more than twice as likely to engage in unsafe sexual practices compared to female students. This finding aligns with evidence from a systematic review in Ethiopia showing higher risk-taking behavior among male youths (20). Gender norms that encourage sexual experimentation among males while discouraging similar behavior among females may partly explain this difference.

Age was also a significant predictor; students aged 18 years or younger were less likely to practice unsafe sexual behaviors compared to older students. This finding is consistent with studies from South Africa, where older adolescents were more likely to engage in risky sexual practices (23). Increased autonomy, exposure to substances, and peer influence among older adolescents may contribute to this pattern.

Behavioral factors, including watching pornography, alcohol consumption, and khat chewing, were strongly associated with unsafe sexual practice. Students who reported watching pornographic films were more likely to engage in unsafe sexual practices, a finding consistent with pooled evidence from Ethiopia and studies conducted among high school students in Gondar (6,24). Exposure to sexual content may normalize risky behaviors and reduce perceived susceptibility to adverse sexual health outcomes.

Alcohol consumption was one of the strongest predictors of unsafe sexual practice. Students who consumed alcohol were nearly three times more likely to engage in unsafe sexual behaviors, consistent with findings from Gondar, Bahir Dar, and Kigali, Rwanda (17,24,25). Alcohol may impair judgment and decision-making capacity, leading to unprotected intercourse or unintended sexual encounters.

Similarly, khat chewing was significantly associated with unsafe sexual practice, which is supported by studies from Alamata, Dessie town, and Dessie Zuria woreda (14,18). Risk behaviors often cluster together, and khat use may increase susceptibility to other substances, such as alcohol, thereby compounding sexual risk-taking behaviors (26).

HBM-related perceptions were also significantly associated with unsafe sexual practice. Students who perceived low social norms supporting consistent condom use were more likely to engage in unsafe sexual behaviors. Likewise, perceived self-efficacy for condom use showed a significant association, consistent with findings from western Nigeria(21). These findings underscore the importance of enhancing adolescents’ confidence and social support for consistent condom use.

Perceived norms regarding abstinence were also associated with unsafe sexual practice. Students who perceived abstinence as socially acceptable and positive were less likely to engage in unsafe sexual behaviors. In this study, low perceived norms for abstinence—defined by beliefs that premarital sex is common, socially acceptable, or a sign of maturity—were protective against unsafe sexual practice. This suggests that adolescents who critically perceive peer norms may resist pressure to engage in risky sexual behaviors. Similar findings have been reported elsewhere, highlighting the role of perceived norms in shaping abstinence behaviors (27).

**Strengths and Limitations**

The cross-sectional design limits the ability to establish causal relationships. Data were collected using self-administered questionnaires, which are subject to recall and social desirability bias, particularly for sensitive behaviors such as sexual practices and substance use. Additionally, the absence of interviewer guidance and the inability to apply complex skip patterns may have resulted in missing or misreported information.

Despite these limitations, the study has notable strengths. A large sample size and random sampling technique were used to enhance representativeness. Rigorous data quality control measures were implemented to minimize bias and improve the reliability of the findings. The use of a theory-based framework (HBM) also strengthens the interpretation of behavioral and perceptual factors associated with unsafe sexual practice.

# C**onclusions**

The study found that about two in five students in the setting engaged in unsafe sexual practices. Factors such as sex, age, perceived norms to abstain, constant condom use, self-efficacy for condom use, watching sex films, chewing khat, and drinking alcohol were significantly associated with unsafe sexual practices among adolescents in the study setting. The Gembella town education office, health office, and school communities are recommended to play their role in reducing behavioral factors around the school environment, in-school health education targeted to their age and gender, and reducing the vulnerability of school youths to unsafe sexual practices. Additionally, intensive interventional research is also important to mitigate the consequences of unsafe sexual practices among in-school youths.

# List of abbreviations

**AOR:** Adjusted Odds Ratio, **CI:** Confidence Interval, **COR**: Crude Odds Ratio, **HBM**: health belief model, **SPSS:** Statistical Package for Social Sciences, and **USP:** unsafe sexual practice

# **Ethical approval and consent to participate**

The study was conducted in accordance with the Declaration of Helsinki, and the protocol was approved by the Ethical Review Committee of College of Health Science of Mattu University. A connecting letter with Ref.no. RPG/325/15 written on June 14, 2023 was taken from Mattu University to Gambella town administration and health office. Each study participant was informed about the study’s purpose, data collection methods, anticipated benefit, and risk, and informed consent was obtained prior to data collection. In case of minors (participants under the age of 16) , informed consent to participate in this study was obtained from the parents or legal guardian/next of kin and assent was obtained from the participants. Privacy and confidentiality were maintained throughout the data collection, analysis, and result dissemination processes.

# Consent for publication: Not applicable

# Availability of data and materials

The datasets used during the current study are available and can be accessed from the corresponding author upon reasonable request.

# Competing interests

The authors declare that they have no competing interests.

**Funding**

The authors declare that there was no funding source for this research or for the publication of this article. The study was conducted, and all the authors are based in a low-income East African country, Ethiopia.

# Author’s contributions

TF and DOD have designed the survey, trained the research team, oversaw the fieldwork, and participated in drafting the manuscript. TF, DOD, AZ, and BN participated in the design of the survey, approved the survey, and oversaw the critical revision of the manuscript. All authors read and approved the final version of the manuscript.

# Acknowledgments

The authors are thankful to Mattu University's Department of Public Health for their support, study participants, data collectors, supervisors, and the Gambella town education office for providing essential information and cooperation.

**Authors’ information**

^a^ Department of Public Health, College of Health Science, Mattu University, Mattu, Ethiopia.^b^ Department of Health Informatics, College of Health Science, Mattu University, Mattu, Ethiopia.^c^ Department of Midwifery, College of Health Science, Mattu University, Mattu, Ethiopia

**ORCID IDs**

Asrat Zewdie: <https://orcid.org/0000-0001-9526-839X>

Dereje Oljira Donacho : <https://orcid.org/0000-0001-6145-2914>

# REFERENCES

1. Carvajal-Velez L, Requejo JH, Ahs JW, Idele P, Adewuya A, Cappa C, et al. Increasing data and understanding of adolescent mental health worldwide: UNICEF’s measurement of mental health among adolescents at the population level initiative. J Adolesc Heal. 2023;72(1):S12–4.

2. Sychareun V, Thomsen S, Chaleunvong K, Faxelid E. Risk perceptions of STIs/HIV and sexual risk behaviours among sexually experienced adolescents in the Northern part of Lao PDR. BMC Public Health. 2013;13(1):1126.

3. Organization WH. The adolescent health indicators recommended by the Global Action for Measurement of Adolescent Health: Guidance for monitoring adolescent health at country, regional and global levels. World Health Organization; 2024.

4. Melesse DY, Mutua MK, Choudhury A, Wado YD, Faye CM, Neal S, et al. Adolescent sexual and reproductive health in sub-Saharan Africa: who is left behind? BMJ Glob Heal. 2020;5(1):e002231.

5. Charbit Y, Omrane M. Introduction: The Vulnerability of Adolescence in Sub-Saharan Africa. In: Gender Inequalities and Vulnerability of sub-Saharan Adolescents: The Case of Benin. Springer; 2023. p. 1–41.

6. Muche AA, Kassa GM, Berhe AK, Fekadu GA. Prevalence and determinants of risky sexual practice in Ethiopia: Systematic review and Meta-analysis. Reprod Health. 2017;14(1):113.

7. Fentahun N, Mamo A. Risky sexual behaviors and associated factors among male and female students in Jimma Zone preparatory schools, South West Ethiopia: comparative study. Ethiop J Health Sci. 2014;24(1):59–68.

8. Arruda EPT, Brito LGO, Prandini TR, Lerri MR, Dos Reis RM, Barcelos TMR, et al. Sexual practices during adolescence. Rev Bras Ginecol e Obs Gynecol Obstet. 2020;42(11):731–8.

9. Conner M, Norman P. Predicting and changing health behaviour: A social cognition approach. Predict Chang Heal Behav Res Pract with Soc Cogn Model. 2015;3:1–29.

10. Downing-Matibag TM, Geisinger B. Hooking up and sexual risk taking among college students: A health belief model perspective. Qual Health Res. 2009;19(9):1196–209.

11. Joseph K. Input Factors That Improve Education Quality In Gambella Town Secondary School Of Gambella. Ambo University; 2022.

12. Girmay A, Mariye T. Risky sexual behavior practice and associated factors among secondary and preparatory school students of Aksum town, northern Ethiopia, 2018. BMC Res Notes. 2019;12(1):698.

13. Mersha A, Teji K, Darghawth R, Gebretsadik W, Shibiru S, Bante A, et al. Risky sexual behaviors and associated factors among preparatory school students in Arba Minch town, Southern Ethiopia. J Public Heal Epidemiol. 2018;10(12):429–42.

14. Mazengia F, Worku A. Age at sexual initiation and factors associated with it among youths in North East Ethiopia. Ethiop J Heal Dev. 2009;23(2).

15. Salih NA, Metaferia H, Reda AA, Biadgilign S. Premarital sexual activity among unmarried adolescents in northern Ethiopia: a cross-sectional study. Sex Reprod Healthc. 2015;6(1):9–13.

16. Alamrew Z, Bedimo M, Azage M. Risky sexual practices and associated factors for HIV/AIDS infection among private college students in Bahir Dar City, Northwest Ethiopia. Int Sch Res Not. 2013;2013(1):763051.

17. Ndagijimana E, Biracyaza E, Nzayirambaho M. Risky sexual behaviors and their associated factors within high school students from Collège Saint André in Kigali, Rwanda: An institution-based cross-sectional study. Front Reprod Heal. 2023;5:1029465.

18. Abay M, Endgashet S, Etana B, Nguse K. Magnitude of premarital sex and associated factors among preparatory school students in Alamata town, North Ethiopia, 2014. Res Rev AJ Heal Prof. 2019;6(2):12–20.

19. Negeri EL. Assessment of risky sexual behaviors and risk perception among youths in Western Ethiopia: the influences of family and peers: a comparative cross-sectional study. BMC Public Health. 2014;14(1):301.

20. Muche AA, Kassa GM, Berhe AK, Fekadu GA. Prevalence and determinants of risky sexual practice in Ethiopia : Systematic review and Meta-analysis. 2017;

21. Odeigah L, Rasaki SO, Ajibola AF, Hafsat AA, Sule AG, Musah Y. High risk sexual behavior among adolescent senior secondary school students in Nigeria. Afr Health Sci. 2019;19(1):1467–77.

22. Srahbzu M, Tirfeneh E. Risky Sexual Behavior and Associated Factors among Adolescents Aged 15‐19 Years at Governmental High Schools in Aksum Town, Tigray, Ethiopia, 2019: An Institution‐Based, Cross‐Sectional Study. Biomed Res Int. 2020;2020(1):3719845.

23. Odimegwu CO, Ugwu NH. A multilevel mixed effect analysis of neighbourhood and individual level determinants of risky sexual behaviour among young people in South Africa. Reprod Health. 2022;19(1):119.

24. Kasahun AW, Yitayal M, Girum T, Mohammed B. Risky sexual behavior and associated factors among high school students in Gondar City, Northwest Ethiopia. 2017;

25. Amare H, Azage M, Negash M, Getachew A, Desale A, Abebe N. Risky sexual behavior and associated factors among adolescent students in Tana Haik high school, Bahir Dar, Northern Ethiopia. Int J HIV/AIDS Prev Educ Behav Sci. 2017;3(4):41–7.

26. Bete T, Lami M, Negash A, Eyeberu A, Birhanu A, Berhanu B, et al. Current alcohol, tobacco, and khat use and associated factors among adults living in Harari regional state, eastern Ethiopia: a community-based cross-sectional study. Front Psychiatry. 2022;13:955371.

27. Selvan MS, Ross MW, Kapadia AS, Mathai R, Hira S. Study of perceived norms, beliefs and intended sexual behaviour among higher secondary school students in India. AIDS Care. 2001;13(6):779–88.


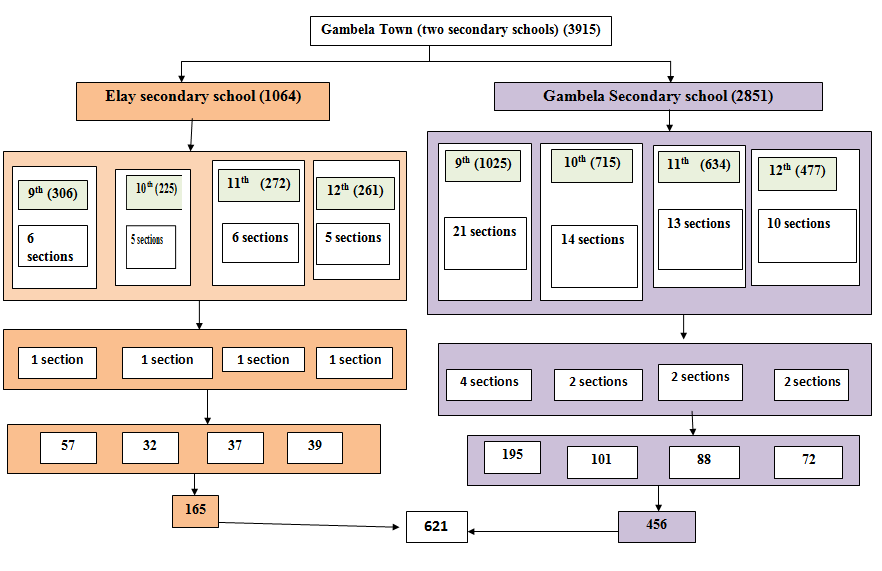
Figure 1: Schematic presentation of sampling procedures to assess unsafe sexual practice, and associated factors among adolescents in secondary schools, Gambella town, Ethiopia, 2023

# Table 1: Socio-demographic characteristics and risky behaviors among adolescents in secondary schools, Gambela, Ethiopia, 2023

| Variables | Categries | Frequency | Percentages |
| --- | --- | --- | --- |
| Age category | 15-19  20-24 | 219  402 | 35.3  64.7 |
| Sex | Male  Female | 261  360 | 42  58 |
| Grade | 9^th^  10^th^  11^th^  12^th^ | 252  133  125  111 | 40.6  21.4  20.1  17.9 |
| Residence | Urban  Rural | 541  80 | 87.1  12.9 |
| Living Arrengements | With family  With relatives  Rented house with peers | 452  104  65 | 72.8  16.7  1.5 |
| Father’s occupation | Farmer  Day laborer  Employed worker  Has own business | 152  121  200  148 | 24.5  19.5  32.2  23.8 |
| Mother’s occupation | Housewife  Daily laborer  Employed worker  Has own business | 261  82  139  139 | 42  13.2  22.4  22.4 |
| Watched sex film in the last 12 months | Yes  No | 231  390 | 37.2  62.8 |
| Chewed khat in the last 12 months | Yes  No | 118  503 | 19  81 |
| Drunk alcohol in the last 12 months | Yes  No | 145  476 | 23.3  76.7 |

# Table 2: Perceived susceptibility to HIV/AIDS and perceived severity of HIV/AIDS among adolescents in secondary schools, Gambella Town, Ethiopia, 2023

| Variable | | Response | Frequancy(%) |
| --- | --- | --- | --- |
| Perceived susceptibility | HIV can be transmitted through sexual intercourse. | Yes | 577 (92.9) |
|  |  | No | 44 (7.1) |
|  | Having sexual intercourse with multiple sexual partners can increase exposure to risk of getting HIV infection. | Yes | 546 (87.9) |
|  |  | No | 75 (12.1) |
|  | Percieved benefits of abstinence as safer sexual practice | Yes | 501 (80.7) |
|  |  | No | 120 (19.3) |
|  | Percieved benefits of condom use as safer sexual practice | Yes | 536 (86.3) |
|  |  | No | 85 (13.7) |
|  | Percieved benefits of limiting sexual partners (being in mutual monogamy) as safer sexual practice | Yes | 435 (70) |
|  |  | No | 186 (30) |
|  | A healthy looking person can have HIV infections in his/her blood. | Yes | 423 (68.1) |
|  |  | No | 198 (31.9) |
|  | If you know a person very well and you trust him/her, you do not have to use condoms when you have sex with him/her. | Yes | 226 (36.4) |
|  |  | No | 395 (63.6) |
|  | People who are in monogamous relation are not at risk for HIV infection, so that, they do not need to use condoms. | Yes | 249 (40.1) |
|  |  | No | 372 (59.9) |
|  | Over all perceived susceptibility to HIV/AIDS | High | 503 (81) |
|  |  | Low | 118 (19) |
| Perceived severity of sexual health risk | HIV AIDS has no cure, so that can lead to death | Yes | 470 (75.7) |
|  |  | No | 151(24.3) |
|  | Unwanted pregnancy and unsafe abortion can cause death of women | Yes | 505 (81.3) |
|  |  | No | 116 (18.7) |
|  | Over all perceived severity of HIV/AIDS | High | 412 (66.3) |
|  |  | Low | 209 (33.7) |

# Table 3: Attitude and perceived social norms about safer sexual practice of abstinence and condom use among adolescents attending secondary schools, in Gambella Town, Ethiopia, 2023

| Variable | | Categories | Frequancy | Percentage | Mean value | SD | Overall | |
| --- | --- | --- | --- | --- | --- | --- | --- | --- |
|  |  |  |  |  |  |  | Low | High |
| *Perceived norms towards unsafe sexual practice* | perceived peer norms about premarital sex | SDA | 95 | 15.3 | 2.84 | 1.37 | 374 (60.2%) | 247 (39.8%) |
|  |  | DA | 234 | 37.7 |  |  |  |  |
|  |  | N | 89 | 14.3 |  |  |  |  |
|  |  | A | 83 | 13.4 |  |  |  |  |
|  |  | SA | 120 | 19.3 |  |  |  |  |
|  | premarital sex is acceptable in the society/religion | SDA | 102 | 16.4 | 2.57 | 1.23 |  |  |
|  |  | DA | 276 | 44.4 |  |  |  |  |
|  |  | N | 103 | 16.6 |  |  |  |  |
|  |  | A | 66 | 10.6 |  |  |  |  |
|  |  | SA | 74 | 11.9 |  |  |  |  |
|  | premarital sex is a sign of maturity | SDA | 108 | 17.4 | 2.61 | 1.26 |  |  |
|  |  | DA | 261 | 42.0 |  |  |  |  |
|  |  | N | 97 | 15.6 |  |  |  |  |
|  |  | A | 77 | 12.4 |  |  |  |  |
|  |  | SA | 78 | 12.6 |  |  |  |  |
| *Attitude towards consistent use of condom* | condom use reduces sexual pleasure | SDA | 68 | 11 | 3.12 | 1.21 | 293 (47.2%) | 328 (52.8%) |
|  |  | DA | 113 | 18.2 |  |  |  |  |
|  |  | N | 222 | 35.7 |  |  |  |  |
|  |  | A | 113 | 18.2 |  |  |  |  |
|  |  | SA | 105 | 16.9 |  |  |  |  |
|  | condoms are not effective in preventing pregnancy and STIs. | SDA | 100 | 16.1 | 2.65 | 1.24 |  |  |
|  |  | DA | 260 | 41.9 |  |  |  |  |
|  |  | N | 84 | 13.5 |  |  |  |  |
|  |  | A | 112 | 18.0 |  |  |  |  |
|  |  | SA | 65 | 10.5 |  |  |  |  |
| *Perceived norms to consistant condome use* | Condom use and perceived mistrust | SDA | 72 | 11.6 | 3.03 | 1.25 | 352 (56.7%) | 269 (43.3%) |
|  |  | DA | 188 | 30.3 |  |  |  |  |
|  |  | N | 141 | 22.7 |  |  |  |  |
|  |  | A | 127 | 20.5 |  |  |  |  |
|  |  | SA | 93 | 15 |  |  |  |  |
|  | Partner suspicion towards condom use request | SDA | 81 | 13 | 3.06 | 1.26 |  |  |
|  |  | DA | 175 | 28.5 |  |  |  |  |
|  |  | N | 163 | 26.2 |  |  |  |  |
|  |  | A | 104 | 16.7 |  |  |  |  |
|  |  | SA | 98 | 15.8 |  |  |  |  |
| *Key: SDA: Strongly disagree, DA: Disagree, N: Neutral, A: Agree, and SA: Strongly agree* | | | | | | | | |

# Table 4: Perceived self-efficacy for abstinence and condom use to avoid unsafe sexual practice among adolescents attending secondary schools, in Gambella Town, Ethiopia, 2023

| Variable | | Category | Frequancy | Percentage | Mean value | SD | Overall | |
| --- | --- | --- | --- | --- | --- | --- | --- | --- |
|  |  |  |  |  |  |  | **Low** | **High** |
| *Perceived self-efficacy for abstinence* | refusal of sexual intercourse | SDA | 95 | 15.3 | 3.8 | 1.49 | 277 (44.6%) | 344 (55.4%) |
|  |  | DA | 32 | 5.2 |  |  |  |  |
|  |  | N | 98 | 15.8 |  |  |  |  |
|  |  | A | 72 | 11.6 |  |  |  |  |
|  |  | SA | 324 | 52.2 |  |  |  |  |
|  | abstinence self efficacy | SDA | 69 | 11.1 | 3.58 | 1.38 |  |  |
|  |  | DA | 67 | 10.8 |  |  |  |  |
|  |  | N | 163 | 26.2 |  |  |  |  |
|  |  | A | 80 | 12.9 |  |  |  |  |
|  |  | SA | 242 | 39 |  |  |  |  |
| *Perceived self-efficacy for condom use* | ability of negotiation for condom use | SDA | 77 | 12.4 | 3.41 | 1.35 | 388 (54.4%) | 283 (45.6%) |
|  |  | DA | 66 | 10.6 |  |  |  |  |
|  |  | N | 200 | 32.2 |  |  |  |  |
|  |  | A | 80 | 12.9 |  |  |  |  |
|  |  | SA | 198 | 31.9 |  |  |  |  |
|  | partener persuasion for condom use | SDA | 71 | 11.4 | 3.51 | 1.32 |  |  |
|  |  | DA | 59 | 9.5 |  |  |  |  |
|  |  | N | 168 | 27.1 |  |  |  |  |
|  |  | A | 130 | 20.9 |  |  |  |  |
|  |  | SA | 193 | 31.1 |  |  |  |  |
|  | consistent condom use | SDA | 95 | 15.3 | 3.30 | 1.42 |  |  |
|  |  | DA | 82 | 13.2 |  |  |  |  |
|  |  | N | 177 | 28.5 |  |  |  |  |
|  |  | A | 78 | 12.6 |  |  |  |  |
|  |  | SA | 189 | 30.4 |  |  |  |  |
| *Key: SDA: Strongly disagree, DA: Disagree, N: Neutral, A: Agree, and SA: Strongly agree* | | | | | | | | |

# Table 5: Unsafe sexual practice among adolescents attending secondary schools, in Gambella Town, Ethiopia, 2023

| Variables | Categories | Frequancy | Percentages |
| --- | --- | --- | --- |
| Ever had sexual intercourse | Yes  No | 251  370 | 40.4  59.6 |
| Own decision to have sex for the first time (n=251) | Yes  No | 154  97 | 61.4  38.6 |
| Reason to have sexual intercourse in the first sexual contact  (n=97) | Alcohol use  Kchat chewing  Economic reason  Peer presure  Partner reinforcement | 27  27  18  16  9 | 27.8  27.8  18.6  16.5  9.3 |
| Condom used during this first time you had sexual intercourse (n=251) | Yes  No | 140  111 | 55.8  44.2 |
| Had sexual intercourse in the last 12 months (n=251) | Yes  No | 227  24 | 90.4  9.6 |
| Number of sexual partners in last 12 months (n=227) | Only one  Two or more | 124  103 | 54.6  45.4 |
| Condom use in the last time you had sexual intercourse* (n=227) | Yes  No | 110  117 | 48.5  51.5 |
| Unsafe sexual practice** (n=621) | Yes  No | 227  394 | 36.6  63.4 |
| Key: * The frequency of condom use was'sometimes' for all of them (110) who reported using condoms.  **If ever had sexual intercourse and had sexual intercourse in the last 12 months and condom unprotected sex, then Yes; otherwise, No | | | |

# Table 6: Multivariable analysis factors associated with unsafe sexual practices among adolescents attending secondary schools in Gambela Town, Ethiopia, 2023

| Varaibles | Categories | Unsafe sexual practice | | Crude OR | Adjusted OR | |
| --- | --- | --- | --- | --- | --- | --- |
|  |  | Yes | No | COR [95% C.I] | AOR [95% C.I] | P-Value |
| Sex | Male | 129 | 132 | 2.61 [1.87-3.65] | 2.38 [1.61-3.51] | <0.001* |
|  | Female | 98 | 262 | 1 | 1 |  |
| Residence | Urban | 192 | 349 | 0.70 [0.44-1.138] | 0.95 [0.55-1.65] | 0.868 |
|  | Rural | 35 | 45 | 1 | 1 |  |
| Age category | Less than 18 | 58 | 161 | 0.49 [0.34-0.712] | 0.65 [0.43-0.97] | 0.038* |
|  | 18 and above | 169 | 233 | 1 | 1 |  |
| Percieved Susseptablity | High | 194 | 309 | 1.62 [1.04-2.51] | 1.49 [0.90-2.47] | 0.120 |
|  | Low | 33 | 85 | 1 | 1 |  |
| Percieved severity of HIV/AIDs | High | 141 | 271 | 0.74 [0.53-1.05] | 1.04 [0.70-1.56] | 0.822 |
|  | Low | 86 | 123 | 1 | 1 |  |
| Percieved Benefit of safe sexual practice | High | 205 | 341 | 1.45 [0.856-2.45] | 1.54 [0.86-2.79] | 0.146 |
|  | Low | 22 | 53 | 1 | 1 |  |
| Perceived norms to abstainance | Low | 115 | 259 | 0.53 [0.38-0.74] | 0.60 [0.41-0.89] | 0.013* |
|  | High | 112 | 135 | 1 | 1 |  |
| Attitude to concestant condome use | Low | 99 | 194 | 0.79 [0.57-1.07] | 1.17 [0.79-1.72] | 0.436 |
|  | High | 128 | 200 | 1 | 1 |  |
| Perceived norms to concestant condome use | Low | 149 | 203 | 1.79 [1.28-2.52] | 2.05 [1.39-3.03] | <0.001* |
|  | High | 78 | 191 | 1 | 1 |  |
| Perceived self-efficacy for condom use | Low | 109 | 229 | 0.66 [0.48-0.92] | 0.59 [0.40-0.86] | 0.007* |
|  | High | 118 | 165 | 1 | 1 |  |
| Watch sex film | Yes | 114 | 117 | 2.39 [1.70-3.35] | 1.50 [1.01-2.20] | 0.047* |
|  | No | 113 | 277 | 1 | 1 |  |
| Chew khat | Yes | 69 | 49 | 3.07 [2.04-4.64] | 2.53 [1.57-4.07] | <0.001* |
|  | No | 158 | 345 | 1 | 1 |  |
| Drink alcohol | Yes | 83 | 62 | 3.09 [2.105-4.53] | 2.93 [1.86-4.62] | <0.001* |
|  | No | 144 | 332 | 1 | 1 |  |

*Key: *siginficant at p-value less than 0.05*
